# Computational Fluid Dynamics Reveals Mass Transfer Limitations in a Pilot‐Scale Microbial Electrolysis Cell

**DOI:** 10.1002/wer.70452

**Published:** 2026-06-19

**Authors:** Oscar Guerrero‐Sodric, Rholand Jordi Navarro‐Quispe, Martí Cortada‐García, Juan Antonio Baeza, Albert Guisasola

**Affiliations:** ^1^ GENOCOV, Department of Chemical, Biological and Environmental Engineering, School of Engineering Universitat Autònoma de Barcelona Bellaterra Spain

**Keywords:** ANSYS Fluent, computational fluid dynamics (CFD), microbial electrolysis cell (MEC), pilot plant, wastewater treatment

## Abstract

The scalability of microbial electrochemical technologies (METs), particularly microbial electrolysis cells (MECs), is constrained by hydrodynamic and mass transfer limitations that hinder efficient resource recovery from wastewater. This study presents a comprehensive computational fluid dynamics (CFD) model of a pilot‐scale MEC (1 m^3^), representing the largest MEC modeled to date and integrating fluid dynamics with bioelectrochemical substrate consumption. The model simulates the spatial distribution of the anolyte under various operational conditions to identify flow‐induced limitations in substrate transport to anodic biofilms. Dead zones and preferential flow paths caused significant inefficiencies, resulting in poor acetate removal under laminar flow. When evaluating the influence of HRT, reaction kinetics, and diffusivity on MEC performance, simulations further underscore that reactor performance is predominantly governed by external mass transfer rather than intrinsic reaction kinetics. To mitigate transport limitations without reducing the volumetric treatment capacity, a recirculation strategy was implemented, enhancing acetate removal efficiency from 16% to 48%. Model predictions agreed well with experimental data from similar pilot‐scale MECs, supporting the validity of the approach. This work is intended as a reduced‐order framework to diagnose hydrodynamic and external mass transfer limitations in large‐scale cassette‐type MECs, offering practical insights for improving reactor design and operation.

## Summary

- The largest spatially resolved microbial electrolysis cell (MEC) model to date was developed.
- The CFD model links hydrodynamics, substrate transport, and anodic consumption.
- Pilot‐scale MEC performance was mainly limited by external mass transfer, not kinetics.
- Flow maldistribution created dead zones and underused anodic surface area.
- Recirculation enhanced substrate access and increased acetate removal from 16% to 48%.

## Graphical abstract

A computational fluid dynamics (CFD) model of a 1‐m^3^ microbial electrolysis cell (MEC) reveals that pilot‐scale MEC performance is governed by hydrodynamics and external mass transfer rather than intrinsic bioelectrochemical kinetics. Recirculation mitigates limitations in external mass transfer, increasing acetate removal from ~16% to ~48%.

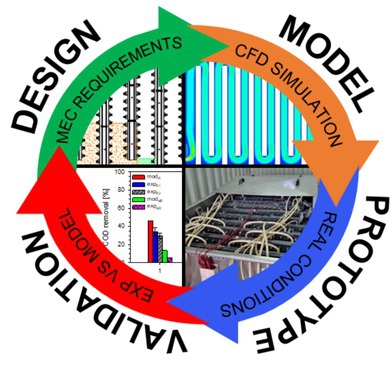

## 1 Introduction

The increasing severity of climate change, together with resource depletion and environmental degradation, highlights the need for sustainable energy technologies. Wastewater treatment is a major but often overlooked contributor to global energy consumption. Conventional wastewater treatment processes consume between 1.3 and 2.1 kJ g^−1^ of chemical oxygen demand (COD) removed, whereas wastewater contains approximately 16.4 kJ g^−1^ COD as internal energy (Dai et al. 2019). Therefore, wastewater treatment processes hold the theoretical potential to become energy self‐sufficient or even net energy producers. As a result, alternative strategies that enable both treatment and energy recovery are receiving increasing attention. Among these, microbial electrochemical technologies (METs) have emerged as a promising option.

METs can simultaneously treat wastewater and recover valuable resources. In particular, microbial fuel cells (MFCs) and microbial electrolysis cells (MECs) convert the chemical energy of organic matter into electricity and hydrogen, respectively. These systems are based on promoting the growth of exoelectrogenic microorganisms capable of transferring electrons generated during their metabolism to external (i.e., solid) electron acceptors such as electrodes. MFCs generate electricity directly by using oxygen as the terminal electron acceptor, whereas MECs require an external energy input to drive the hydrogen evolution reaction (HER) at the cathode, offering a more energy‐efficient alternative to conventional water electrolysis (Rozendal et al. 2007). Despite their potential, scaling up METs from laboratory to industrial applications remains a critical challenge.

Most MET studies have been conducted at laboratory scale (< 1 L), where systems are typically well mixed. At larger scales, however, hydrodynamic mass transfer limitations become dominant due to the need to incorporate high‐surface‐area electrodes in a bioreactor (Corona‐Martínez et al. 2025). Nonuniform flow distributions lead to preferential pathways and stagnant zones, reducing substrate availability to electroactive biofilms and lowering both current density and energy recovery efficiency (Delattre et al. 2020; Lacroix et al. 2021). In addition, the high cost of key components such as electrodes and ion exchange membranes further limits the economic feasibility of large‐scale systems (Aiken et al. 2019). Therefore, improving reactor design and operation is essential for successful scale‐up.

To address these challenges, mathematical modeling has emerged as an essential tool for guiding MET development and scaling up. Whereas early MET models have primarily focused on microbial metabolism, biofilm growth, and electrochemical properties at laboratory scale (Gadkari et al. 2019; Marcus et al. 2007, among many others), they often neglect key fluid dynamics effects that become critical at pilot and industrial scales (Leicester, Amezaga, and Heidrich 2020; Leicester, Amezaga, et al. 2020).

Computational fluid dynamics (CFD) can provide detailed insights into flow behavior, mass transport, and reaction kinetics within METs. Understanding the interactions between biofilm activity and fluid flow is particularly important at pilot scale, where spatial variations in velocity and substrate concentration can lead to heterogeneous biofilm activity and performance losses (Hernández‐García et al. 2020).

Significant advances have been made in applying CFD to MFCs. For instance, Michie et al. (2014) demonstrated that helical anode geometries considerably affected hydrodynamic conditions, with elevated shear rates enhancing both biofilm development and power density. Kim et al. (2014) provided a systematic CFD‐based evaluation of 12 anode configurations, revealing that internal structural design governs system performance, fluid flow characteristics, and theoretical energy output. Similarly, Vilà‐Rovira et al. (2015) highlighted the critical role of flow regime in substrate transport and biofilm growth, establishing CFD as a predictive tool for optimizing electrode–fluid interactions.

Despite these advances, only a few studies have successfully integrated CFD with bioelectrochemical modeling in MECs. For instance, Hernández‐García et al. (2020) simulated MEC hydrodynamics using COMSOL Multiphysics, but reaction kinetics were not dynamically coupled with fluid flow. More recently, Day et al. (2022) developed one of the first models integrating both hydrodynamics and electrochemical reactions in a pilot‐scale reactor using Python, yet large‐scale applications remain largely unexplored. Therefore, there is a need for computational models that capture the complexity of large‐scale MEC systems while remaining computationally efficient. This constitutes a key knowledge gap in MEC scale‐up, as the relative importance of hydrodynamics, substrate transport, and intrinsic reaction kinetics at pilot scale remains insufficiently understood. Addressing this gap is essential for developing design and operational strategies that improve reactor performance while maintaining practical scalability.

The present study aims to develop a spatially resolved CFD model of a pilot‐scale MEC (1 m^3^) that integrates both fluid dynamics and bioelectrochemical substrate consumption. This work presents the largest MEC modeled to date and introduces a reduced‐order model aimed at diagnosing hydrodynamic and external mass transfer limitations. The key objectives were (i) to characterize flow patterns and substrate distribution within the reactor, with particular attention to mass transfer limitations at the anode interface, (ii) to investigate the influence of key parameters, including hydraulic retention time (HRT), substrate diffusivity, reaction kinetics, and flow recirculation, on reactor performance, and (iii) to identify design and operational strategies that enhance substrate availability and improve overall reactor efficiency. This framework is intended to support the optimization and scale‐up of MEC systems using CFD tools, such as ANSYS Fluent.

## 2 Methodology

### 2.1 Computational Domain

**FIGURE 1:**
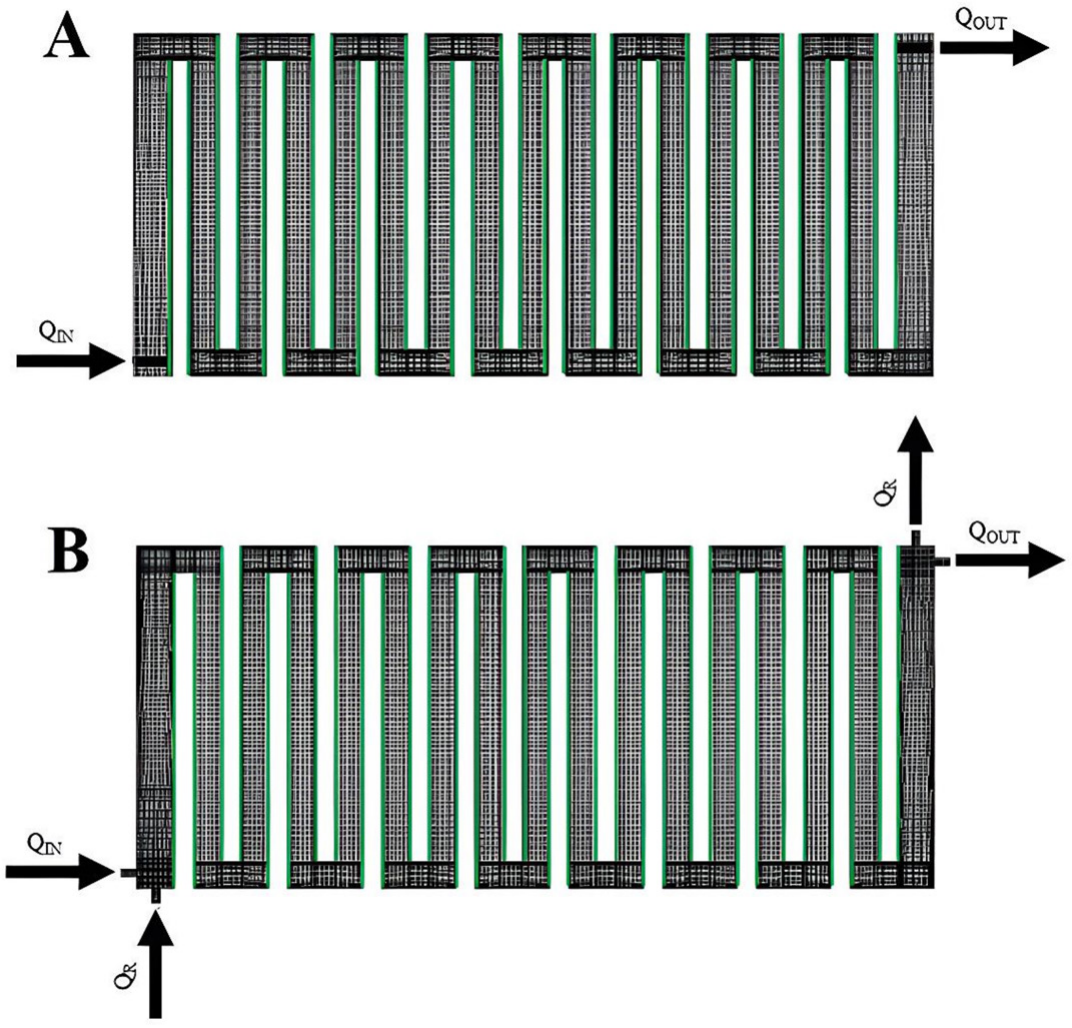
Computational mesh of the MEC reactor model: (A) without recirculation and (B) with recirculation. Green boundaries correspond to the outer surfaces of the cassettes, where exoelectrogenic biofilm is assumed to develop. Arrows indicate flow direction for influent (Q_IN_) and effluent (Q_OUT_), and the recirculation flow (Q_R_) is shown in (B).

**FIGURE 2:**
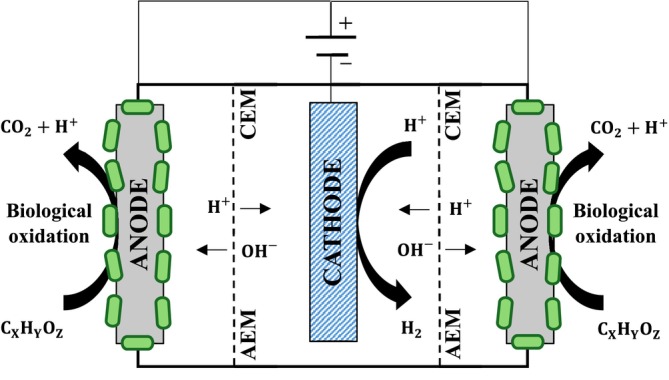
Schematic of a cassette‐type MEC, showing the arrangement of two anodes and a central cathode, interconnected by a power source and separated by a pair of anion exchange membranes (AEMs) or cation exchange membranes (CEMs).

The computational domain consisted of a two‐dimensional (2D) reactor model in which 16 cassette modules, each working as a double‐chamber MEC, are arranged vertically, allowing wastewater to flow through the spaces between them. The anodes face the wastewater, and the cathode is contained in another chamber that is separated by a pair of ion exchange membranes, accounting for 32 anodes and 16 cathodes in total (Figures 1 and 2). This design was adapted from the original concept proposed by Heidrich et al. (2013) and further modified in Baeza et al. (2017) and Guerrero‐Sodric et al. (2023, 2024). Particularly, the present model represents an existing 1‐m^3^ MEC pilot‐scale reactor that the research group developed for the European LIFE+ NIMBUS project (Guerrero‐Sodric et al. 2025). This large‐scale prototype was designed, built, and operated to produce hydrogen from the primary effluent of a large wastewater treatment plant (Barcelona, Spain). Detailed information about the prototype can be found in the Supporting Information (Section S2).

The computational grid was discretized using square cells with mesh refinement applied at regions with expected high gradients in velocity or substrate concentration, such as near the walls and bends of the reactor. The final mesh, consisting of 57,096 cells, was designed based on a previous mesh screening study to ensure a balance between computational efficiency and accuracy (Cortada‐Garcia et al. 2018). Different optimization strategies to enhance external mass transfer in the system were explored. One of the strategies involved applying a recirculation flow of the wastewater (anolyte). For this study, a finer mesh was used, and an extra inlet and outlet for recirculation were added (Figure 1B).

### 2.2 CFD Modeling

The simulations were performed using ANSYS Fluent (Ansys Workbench 2022 version) on a computer equipped with a 1.10‐GHz Intel Celeron CPU N4100 processor and 8‐GB RAM. The reactor was represented as a 2D longitudinal domain to capture the main velocity and substrate concentration gradients along the anode chamber while keeping the model complexity consistent with the reduced‐order objective of the study. In ANSYS Fluent, this 2D formulation is treated as a unit‐depth approximation, meaning that variations in the out‐of‐plane direction are not explicitly resolved. The governing equations were discretized over control volumes or computational cells using the finite volume method (FVM), resulting in a set of algebraic equations that express the conservation laws of mass, momentum, and species transport within each cell. This simplification neglects potential three‐dimensional (3D) effects, including transverse flow heterogeneities, side‐wall effects, inlet/outlet maldistribution, secondary flows, and local 3D mixing around the cassettes. These effects may influence local mixing and mass transfer and should therefore be considered in future full 3D simulations or experimental validation studies.

The continuity equation (Equation 1) represents mass conservation for an incompressible fluid.

(1) ∇·u=0

The momentum conservation Navier–Stokes equation (Equation 2) describes fluid dynamics for an incompressible fluid with a velocity $u=\left(u_{x},u_{y},u_{z}\right)$, constant density (*ρ*), and isothermal conditions. It is expressed by density multiplied by velocity, and it integrates pressure gradients (*p*) and viscous stress (*τ*).

(2) $$\frac{\partial \left(\mathrm{\rho u}\right)}{\partial t}=-\nabla \cdot \left(\mathrm{\rho uu}\right)-\nabla p+\nabla \cdot \tau$$

The viscous stress (*τ*) represents the forces exerted by fluid elements on adjacent fluid elements due to their velocity gradients and the fluid's resistance to deformation. Equation (3) shows its calculation for incompressible Newtonian fluids.

(3) τ=2μD

where *D* (Equation 4) stands for the deformation rate tensor for isotropic fluids.

(4) $$D=\frac{1}{2}\left(\nabla u+{\left(\nabla u\right)}^{T}\right)$$

Depending on the operational conditions of the MEC, the flow regime of the anolyte can be laminar or turbulent, which directly influences the formulation of the governing equations. Because the HRTs in the pilot MEC ranged between 1 and 20 days, two approaches were evaluated: A laminar flow model was applied under high HRTs, whereas a turbulent model was implemented for low HRTs. In the laminar cases, the flow was described using the continuity and momentum conservation equations.

Subsequently, when evaluating the effects of implementing recirculation, only a turbulent flow model was used. ANSYS Fluent offers several turbulence models, including Spalart‐Allmaras, k‐ε (Standard, RNG, Realizable), k‐ω (Standard, SST), Transition k‐kl‐ω, Transition SST, and Reynolds Stress Model (RSM). Given computational considerations and flow characteristics, the Realizable k‐ε turbulence model was selected.

Under turbulent conditions, the momentum Equation (2) was solved in Reynolds‐averaged form to account for the effect of velocity fluctuations through the Reynolds stress tensor (Equation 5):

(5) $$\frac{\partial \left(\rho \bar{u}\right)}{\partial t}=-\nabla \cdot \left(\rho \bar{u}\bar{u}\right)-\nabla \left(\rho \bar{u^{\prime }u^{\prime }}\right)-\nabla p+\mu \nabla \cdot \left(\nabla \bar{u}\right)$$

In this formulation, the instantaneous velocity is decomposed into a time‐averaged component and a fluctuating component. Therefore, a Reynolds decomposition is employed, expressing velocity as the sum of a mean velocity ($\bar{u}$) and a fluctuating velocity component ($u^{\prime }$) (Equation 6):

(6) $$u=\bar{u}+u^{\prime }$$

To address the Reynolds stress terms, the Boussinesq approximation is employed (Equation 7) to represent the stresses for the *x* and *y* directions as follows (this approach can readily be extended to three dimensions):

(7) $$-\left(\rho \bar{u^{\prime }u^{\prime }}\right)=\mu_{t}\left(\frac{\partial \bar{u_{x}}}{\partial y}+\frac{\partial \bar{u_{y}}}{\partial x}\right)-\frac{2}{3}\mathrm{\rho k}\delta_{x,y}$$

where *k* (Equation 8) denotes the turbulent kinetic energy, *δ*_*x*,*y*_ is the Kronecker delta (equal to 1 when *x* = *y* and 0 otherwise), and *μ*_*t*_ (Equation 9) represents the turbulent viscosity.

(8) $$k=\frac{1}{2}\left(\bar{u_{x}^{\prime 2}}+\bar{u_{y}^{\prime 2}}+\bar{u_{z}^{\prime 2}}\right)$$

(9) $$\mu_{t}=\frac{\rho C_{\mu }k^{2}}{\varepsilon }$$

To solve Equation (9), the turbulent kinetic energy and turbulence dissipation rate (*ε*), described by Equations (10) and (11), are needed.

(10) $$\frac{d\left(\mathrm{\rho k}\right)}{\mathrm{dt}}=-\nabla \cdot \left(\mathrm{\rho k}\bar{u}\right)+\nabla \left(\left(\mu +\frac{\mu_{t}}{\sigma_{k}}\right)\nabla k\right)+G_{k}-\rho \varepsilon$$

(11) $$\frac{d\left(\rho \varepsilon \right)}{\mathrm{dt}}=-\nabla \cdot \left(\rho \varepsilon \bar{u}\right)+\nabla \left(\left(\mu +\frac{\mu_{t}}{\sigma_{\varepsilon }}\right)\nabla \varepsilon \right)+\rho C_{1}S_{\varepsilon }-\rho C_{2}\frac{\varepsilon^{2}}{k+\sqrt{\mathrm{v\varepsilon}}}$$

where *G*_*k*_ (Equation 12) represents the generation of turbulent kinetic energy due to the mean velocity gradients, *C*_1_ is a term that depends on *S*, and *S* is the modulus of the mean strain rate tensor.

(12) $$G_{k}=\mu_{t}S^{2};C_{1}=\max\left(0.43,\frac{\eta }{\eta +5}\right);\eta =S\frac{k}{\varepsilon };S=\sqrt{2S_{\mathrm{xy}}S_{\mathrm{xy}}}$$

For this fluid, and under isothermal conditions, the flow was resolved without coupling to the energy equation. Accordingly, the laminar simulations were based on the mass and momentum conservation equations, whereas cases involving recirculation, which increased local velocities and enhanced mixing, were simulated using the Realizable k‐ε turbulence model. This model was selected because of its robustness for wall‐bounded flows and its suitability for representing substrate mixing under the hydraulic conditions evaluated.

### 2.3 MEC Model

To effectively simulate the reactor hydrodynamics while managing computational complexity, a simplified MEC model was implemented. Given computational constraints and the complexity of the reactions occurring within MEC systems, the model was simplified by focusing exclusively on acetate (*A*) as the sole electron donor substrate and considering a single exoelectrogenic bacterial species. This simplification results in a better understanding of acetate consumption profiles and fluid behavior without explicitly modeling biomass dynamics or hydrogen production. Consequently, the simplified reaction modeled is

$$A+M\overset{k_{r}}{\to }P+M$$

where P represents a hypothetical reaction product and M symbolizes the mediator or bacteria, assumed to be permanently active on the anode surface, analogous to a catalyst with unlimited durability. The reaction rate constant for acetate consumption is expressed as *k*_*r*_. It was calculated based on experimental data of a 135‐L MEC reactor treating synthetic and real urban wastewater (Guerrero‐Sodric et al. 2023), resulting in a *k*_*r*_ of 4.80 × 10^−6^ s^−1^.

Furthermore, the following assumptions needed to be applied for simplification:

- Methanogenic acetoclastic bacteria were not considered. Literature highlights their possible competitive interaction with ARB for acetate, potentially reducing reactor efficiency (Montpart et al. 2015; Rago et al. 2017), but its effect was considered negligible in this case because these processes are only favored at acetate concentrations higher than the one expected in the reactor.
- The biofilm was exclusively formed by a single ARB type that was uniformly distributed on the anode surface.
- The biofilm had uniform density and constant metabolic activity across the entire anode surface, eliminating biomass concentration or growth dynamics as limiting factors in substrate consumption (i.e., biofilm is treated as a uniform and constant catalytic surface).
- Mediator (*M*) regeneration was considered instantaneous and infinite, analogous to a catalyst with unlimited availability.
- Despite Monod kinetics being common for microbial substrate consumption, the current model applied linear kinetics as it assumed that the MEC permanently operated at low acetate concentrations (i.e., on the initial linear phase of a Monod model).
- The model assumed isothermal conditions and a homogeneously distributed temperature within the reactor. Thus, *k*_*r*_ and the other physicochemical properties of the fluid in the reactor were considered constant.

These assumptions enable the isolation of hydrodynamic and transport effects, which are the primary focus of this study. However, in real systems, spatial heterogeneities in biofilm distribution, variations in microbial activity, and substrate competition may also affect reactor performance and should be considered in more comprehensive models. It is important to note that this model represents a reduced‐order approach focused on coupling hydrodynamics with substrate transport and consumption. It is not intended to fully describe biofilm dynamics, current generation, or hydrogen production but rather to assess the impact of external mass transfer limitations at pilot scale.

### 2.4 Mass Balance Equations

Substrate transport and consumption within the reactor were modeled using Equation (13):

(13) $$\frac{\partial \rho }{\partial t}Y_{i}=-\nabla \left(\rho Y_{i}u\right)-\nabla J_{i}+R_{I}$$

Here, *Y*_*i*_ represents the mass fraction of acetate relative to the total mass. The term $\nabla \left(\rho Y_{i}u\right)$ is the variation of the substrate due to convection, where u is the fluid velocity obtained from the equations described above. The term $\nabla J_{i}$ is the variation due to diffusion, and $R_{I}$ represents the chemical reaction taking place. The diffusion term differs according to flow regime; under laminar conditions, it follows Fick's law (Equation 14):

(14) $$J_{i}=-\rho D_{\text{acet}}\nabla Y_{i}$$

where $D_{\text{acet}}$ is the diffusion coefficient of acetate in water. For turbulent flows, an additional turbulent Schmidt number (*Sc* = 0.7) is included, modifying the diffusion term as Equation (15):

(15) $$J_{i}=\left(-\rho D_{\text{acet}}+\frac{\mu_{t}}{\mathrm{Sc}}\right)\nabla Y_{i}$$

As mentioned above, surface reactions on the anodes are described using first‐order chemical kinetics (Equation 16):

(16) $$R_{i}=M_{\text{acet}}\gamma \left(k_{r}{\left[C_{\text{acet}}\right]}^{\beta }\right)$$

where $M_{\text{acet}}$ is the molecular weight of acetate, $C_{\text{acet}}$ is the acetate concentration, and γ and β are the stoichiometric coefficient and reaction order, respectively (both set to 1 according to the simplifications outlined previously).

### 2.5 Evaluation of Simulation Results

The performance of the model was assessed based on the acetate removal throughout the reactor. ANSYS Fluent provides outlet acetate concentrations ($C_{\text{out}}$), whereas inlet concentrations ($C_{\text{in}}$) are predefined in the simulation setup. The acetate removal efficiency ($\eta_{\text{elim}}$) was calculated using Equation (17).

(17) $$\eta_{\text{elim}}=\frac{C_{\text{in}}-C_{\text{out}}}{C_{\text{in}}}\cdot 100$$

### 2.6 Simulation Parameters

All simulations were conducted under steady‐state conditions. The inlet acetate concentration was maintained constant at 0.4 kg m^−3^ in all cases, which corresponds to a mass fraction of 4 × 10^−4^ g_acet_ g_T_^−1^ relative to the total mass of liquid. The reaction mechanism was only considered at the wall boundaries defined as cassettes, as illustrated in Figure 1. Additionally, in scenarios involving recirculation, the recirculation flow rate (Q_R_) was set to 0.5–2.5 times the inlet flow rate (0.01661–0.08305 kg s^−1^), consistent with current experimental practices. Table S1 provides a list of the parameters employed in the present model, and Table S2 shows the established boundary conditions.

## 3 Results and Discussion

### 3.1 Investigating Fluid Flow Behavior and Substrate Distribution in the MEC

**FIGURE 3:**
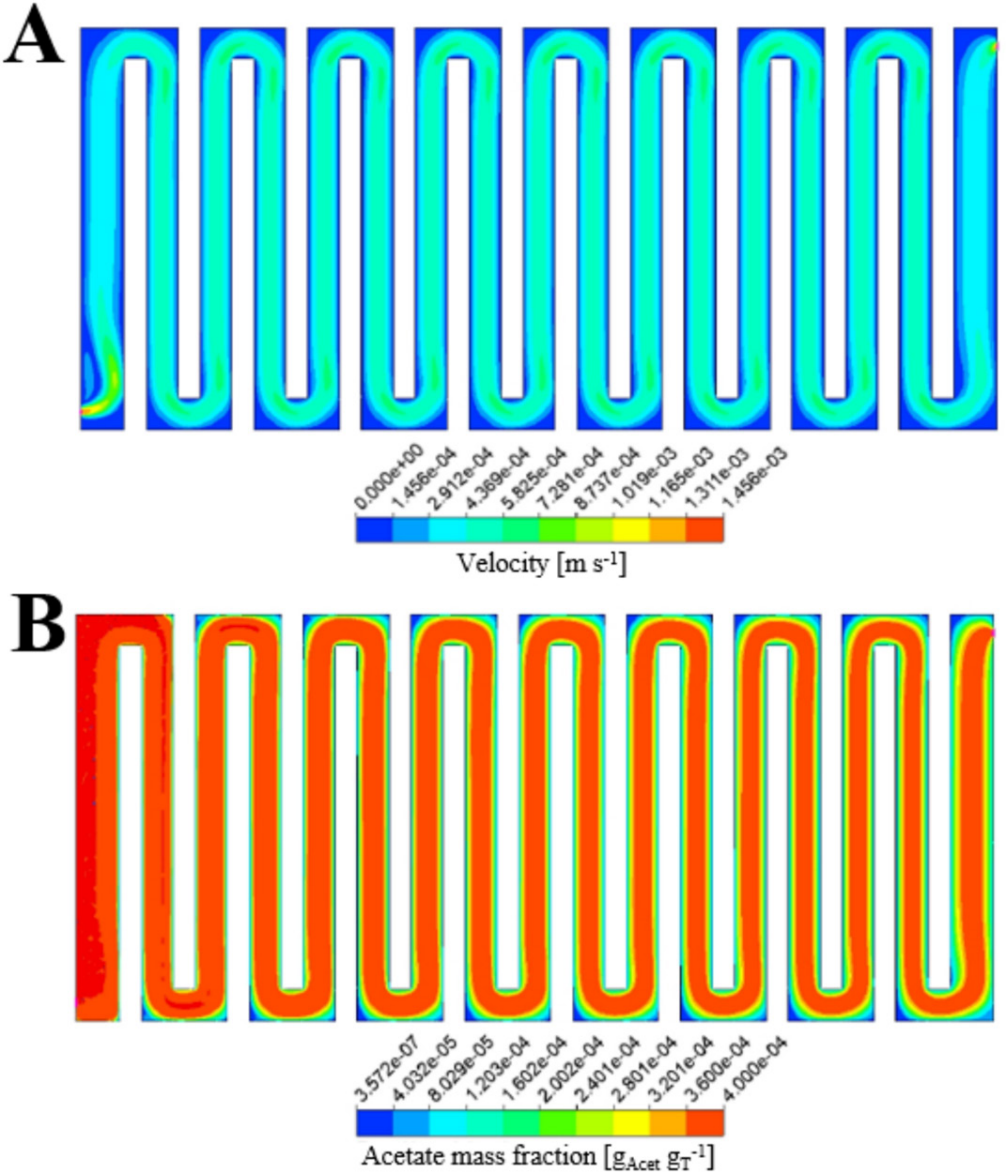
(A) Anolyte velocity profile under base conditions (HRT = 1 day, acetate concentration = 0.4 kg m^−3^), and (B) acetate mass fraction profile under base conditions (HRT = 1 day, acetate concentration = 0.4 kg m^−3^).

Initially, a base scenario with an HRT of 1 day and an influent acetate concentration of 0.4 kg m^−3^ was modeled. Under these conditions, the low Reynolds number of the anolyte <100,Re=ρuLμ−1,L=0.12m indicates predominantly laminar flow. Thus, the mixing between different fluid layers is primarily governed by molecular diffusion rather than convective transport. This results in well‐defined velocity gradients along the channel's width (Figure 3A). Furthermore, the flow rate remains nearly constant along the reactor, with higher velocities at the inlet and outlet regions where the cross‐sectional area narrows down. The model predicts that the central region of the flow channel exhibits moderate velocities, whereas flow in the corners and near the cassette walls is close to stagnant. Specifically, the cross‐sectional analysis of the channel reveals a peak velocity of 5.69 × 10^−4^ m s^−1^ in the center, decreasing to nearly zero at the surface of the cassettes. This pattern indicates suboptimal mixing and the formation of a preferential path that bypasses certain reactor sections. Consequently, the acetate concentration profile (Figure 3B) exhibits similar gradients across the channel width, with the lowest concentrations near the surface of the cassettes.

Exoelectrogens colonizing the surface of the anodes consume the local acetate, and the minimal flow velocity leads to external mass transfer limitations and prevents the delivery of fresh substrate. Therefore, acetate transport from the bulk liquid to the surface of the cassettes relies primarily on diffusion, reducing the overall substrate uptake rate. The combined effects of poor mixing and the short HRT result in a relatively high outlet acetate concentration (0.34 kg m^−3^) and a low removal efficiency of 16%. From a design perspective, this limited efficiency suggests that optimizing reactor hydrodynamics is necessary. This could involve either increasing turbulence in areas of reduced flow or altering the reactor's geometry to foster more uniform velocity and concentration profiles. Simple design modifications, such as strategic positioning of baffles or altering the cassette spacing, could improve convective transport and reduce the formation of dead zones. Additionally, operational strategies involving periodic mixing pulses could help mitigate substrate depletion on the anode surface.

**FIGURE 4:**
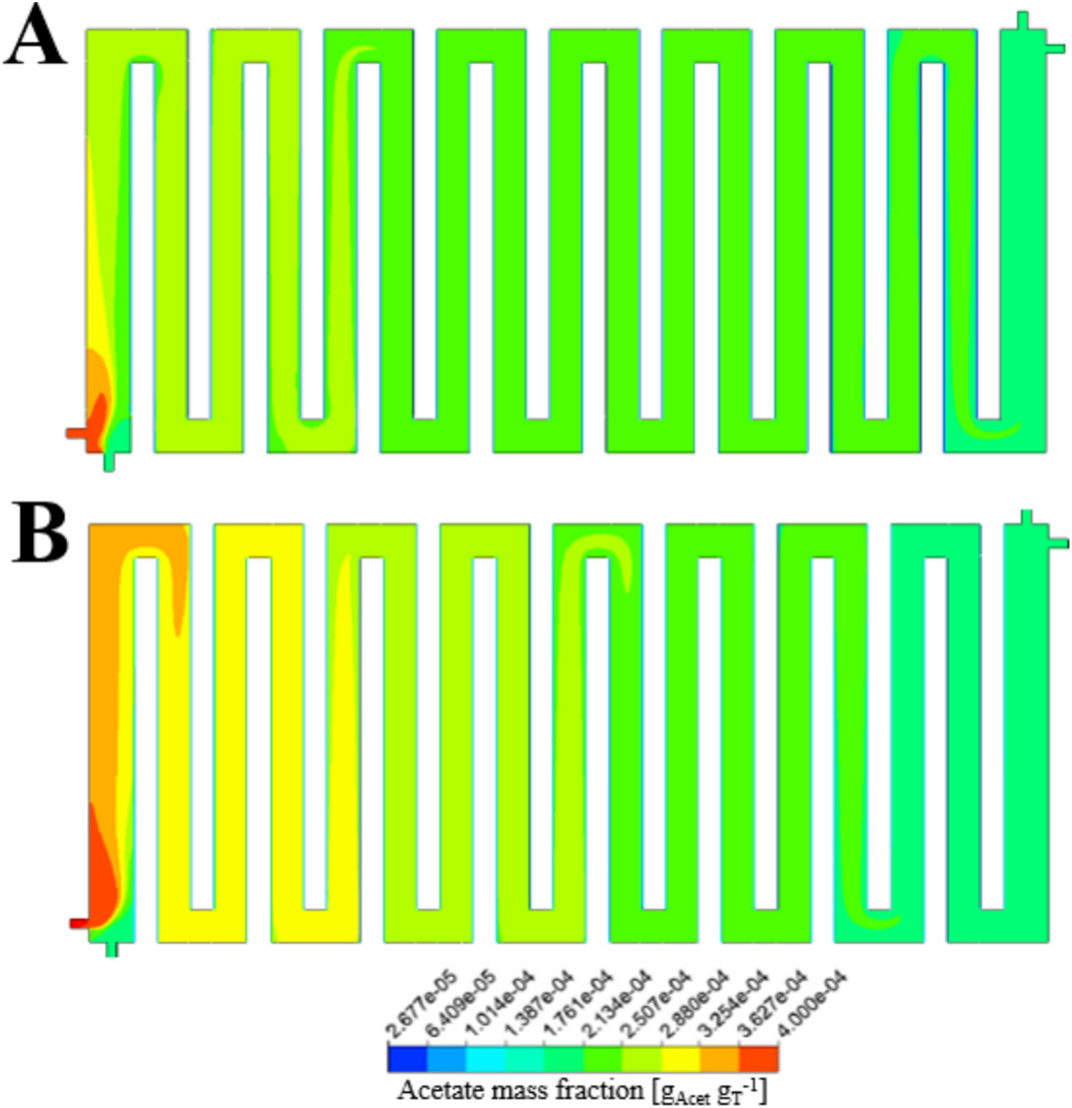
Acetate mass fraction profile under base conditions (HRT = 1 day, acetate concentration = 0.4 kg m^−3^) with recirculation ratios of (A) 2.5 and (B) 0.5.

A common strategy reported in different METs is the inclusion of a substrate recirculation stream that enhances external mass transfer and increases global substrate conversion. Figure 4A shows the acetate concentration profile at an HRT of 1 day and a recirculation ratio of 2.5 (i.e., a recirculated flow rate of 2.5 times the inlet flow rate), which equals 0.08305 kg s^−1^. Under these conditions, although some dead zones still persist in reactor bends, the model predicts a 260% increase in the average velocity of the anolyte compared to the model prediction with no recirculation (from 2.95 × 10^−4^ to 1.06 × 10^−3^ m s^−1^). Furthermore, the predicted outlet acetate concentration is 0.21 kg m^−3^, which translates into a considerable improvement in the removal efficiency (48%): nearly a fourfold increase compared to the simulation with no recirculation at the same HRT (Figure 3B). This improvement is mainly attributed to changes in fluid dynamics near the anode surface. Under laminar conditions, low velocities at the anode limit substrate transport, resulting in diffusion‐controlled regimes. Recirculation increases local flow velocities, reducing the thickness of the concentration boundary layer and thereby enhancing mass transfer to the biofilm. As a result, substrate availability at the anode surface increases, directly contributing to improved acetate removal.

Because pumping energy requirements increase approximately linearly with flow, energy savings were assessed by reducing the recirculation flow rate. When the recirculation ratio is lowered to 0.5, the predicted outlet concentration remains around 0.22 kg m^−3^ (45% removal, Figure 4B), resulting in substantial energy savings with minimal performance loss, as the flow distribution and removal efficiency remain comparable to the higher recirculation scenario. These findings suggest that a recirculation strategy can effectively boost the overall MEC performance by reducing external mass transfer limitations.

Under these operating conditions, the reactor develops a concentration gradient along the anode chamber, with downstream cassettes being exposed to progressively lower acetate concentrations. Consequently, acetate removal, current density, and hydrogen production are lower in these sections than in upstream units. This behavior is consistent with plug‐flow operation and reflects the gradual consumption of substrate along the reactor length. Therefore, improving reactor performance does not necessarily require eliminating this gradient but rather ensuring that all cassettes operate under nonlimiting substrate conditions. In this context, optimizing the location and configuration of recirculation flows may help reduce underutilized regions and improve substrate accessibility, thereby enhancing the overall use of the electroactive surface area.

### 3.2 Sensitivity Analysis of External Mass Transfer Limitations

**FIGURE 5:**
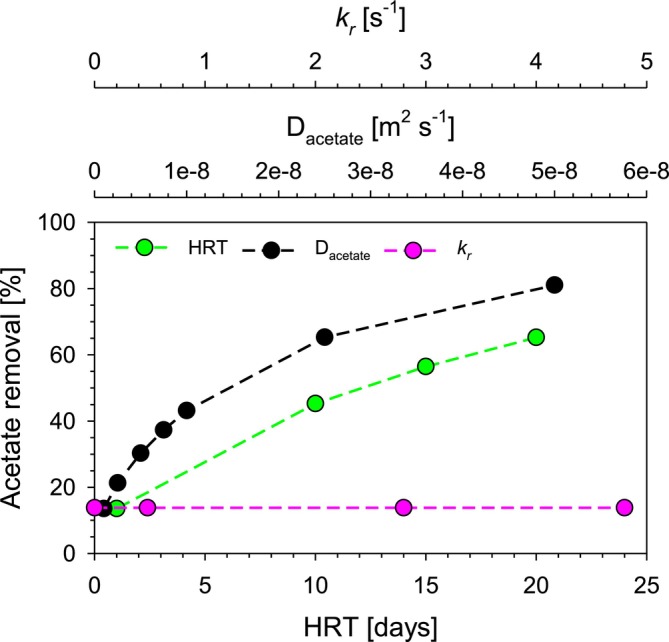
Acetate removal efficiency at different HRTs, kinetic constants (*k*), and acetate diffusivity (*D*_*acet*_).

**FIGURE 6:**
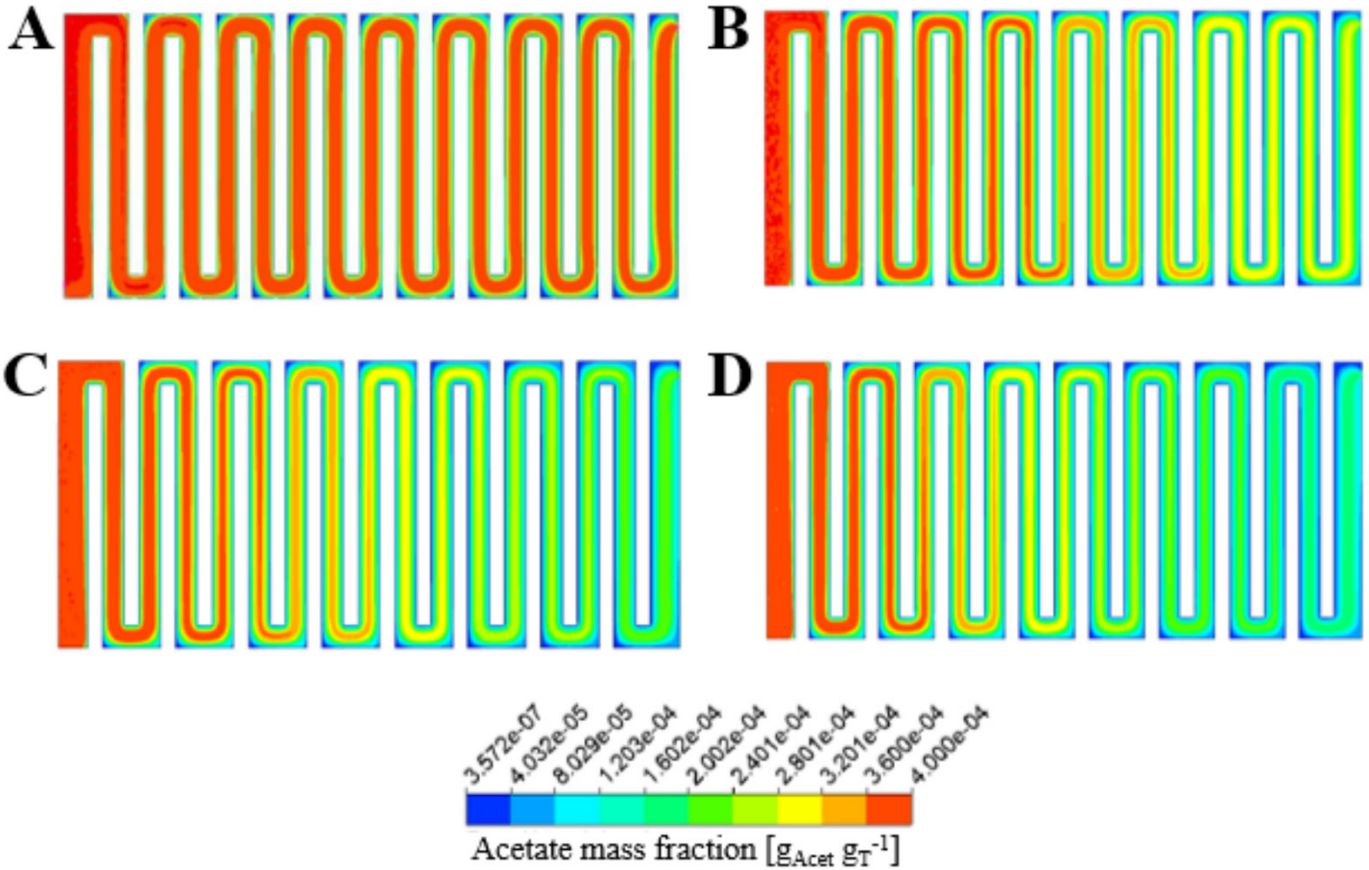
Acetate mass fraction profiles at different HRTs: (A) 1 day, (B) 10 days, (C) 15 days, and (D) 20 days.

The potential external mass transfer limitations in the “no recirculation” scenario were further investigated through a sensitivity analysis in which the HRT, the reaction rate constant (*k_r_*), and acetate diffusivity (*D*_*acet*_) were systematically varied to assess their impact on substrate removal and flow distribution. Firstly, to address the low substrate removal efficiency, the effect of varying the HRT was examined. Increasing the HRT from 1 to 2 days modestly raised the acetate removal efficiency from 16% to 21%, reducing the outlet concentration from 0.34 to 0.32 kg m^−3^. More substantial improvements were observed at higher HRTs (10–20 days), with removal efficiency ranging from 45% to 65% (Figure 5). This underscores the critical influence of HRT on substrate removal, as longer HRTs allow more time for the substrate to diffuse and react at the anode surface (Figure 6). However, while extending the HRT improves removal efficiency, it simultaneously compromises two fundamental key performance indicators of the MEC: (i) It reduces the volumetric treatment capacity, and (ii) it compromises the coulombic efficiency, as substrate competition with other microorganisms (in this case, acetoclastic methanogens that convert acetate into methane) is favored. This trade‐off may pose challenges in industrial applications where maintaining high inlet flow rates is crucial for operational feasibility and cost‐effectiveness.

Acetate diffusivity also had a significant effect on the acetate removal efficiency. Increasing the diffusivity from 1.26 × 10^−9^ to 5.00 × 10^−8^ m^2^ s^−1^ led to a higher acetate removal efficiency than merely extending the HRT. However, in practice, the mass diffusivity of most organic molecules in aqueous solutions is on the order of 10^−9^ m^2^ s^−1^ (Haynes 2016), and substantial increases would require operational adjustments, such as higher temperatures or lower pressures, bringing additional energy costs and practical challenges.

Compared to the HRT and the *D*_*acet*_, the analysis revealed that increasing *k*_*r*_ by several orders of magnitude (from 4.80 × 10^−6^ to 2.16 s^−1^) had a minimal impact on the removal efficiency under the simulated conditions. Because substrate transport from the bulk liquid to the reaction interface is the rate‐determining step, any potential enhancements in intrinsic reaction kinetics are irrelevant in the present model, meaning that acetate cannot reach the biofilm quickly enough for reaction kinetics to exert a significant influence. These results further support the idea that improving external mass transfer, whether through optimized flow conditions or through anodic surface/biofilm distribution, is the most impactful strategy for enhancing system performance, as it contributes to maintaining a more uniform concentration profile, thus reducing the risk of localized depletion zones that could hinder microbial activity and the electrochemical performance of the MEC.

### 3.3 Model Validation Using Experimental Data From Three Pilot‐Scale MECs

To validate the model, we focused on pilot plants previously built and operated by our own research group, as the reactor architecture (i.e., cassette geometry, spacing, and flow distribution) closely matches the system simulated in this study. The available datasets include (i) a 130‐L MEC consisting of 10 cassettes (Baeza et al. 2017) (exp_NR_), (ii) a 1000‐L MEC with 10 cassettes (Guerrero‐Sodric et al. 2025) (exp_R1_), and (iii) a 135‐L MEC with 9 cassettes (Guerrero‐Sodric et al. 2023) (exp_R2_) (Guerrero‐Sodric et al. 2025).

**FIGURE 7:**
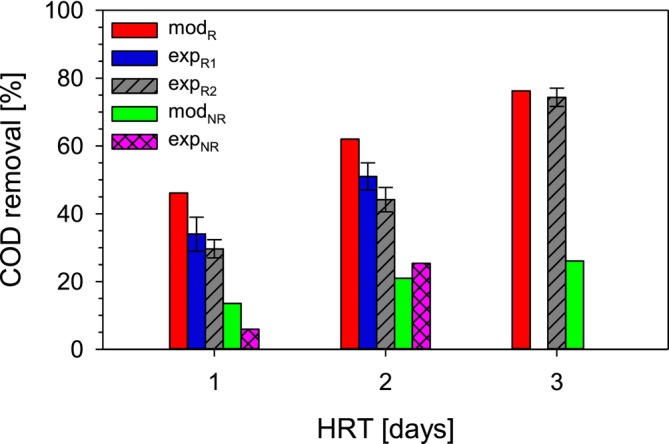
Comparison of COD removal efficiencies among recirculation, no recirculation, and experimental conditions. mod refers to the predicted values by the model, exp refers to the experimental values, R indicates recirculation, and NR indicates no recirculation. Error bars represent standard deviations.

Figure 7 presents a comparison of the predicted and experimental acetate removal efficiencies under operational scenarios with and without recirculation across varying HRTs. For instance, Baeza et al. (2017) operated at an HRT of 2 days and similar influent COD concentrations (0.3–0.5 kg m^−3^), achieving around 25% removal efficiency without recirculation, whereas the present model predicts a similar efficiency (21% at 2 days of HRT) under these conditions. Guerrero‐Sodric et al. (2023) operated at HRTs ranging from 1.1 to 3.9 days and COD concentrations of 0.2–0.3 kg m^−3^, achieving a removal efficiency of 74% ± 3% with recirculation at an HRT of 3 days, in line with the value predicted by the model (76%) under these conditions.

Although certain discrepancies between the experimental and predicted values are evident, the overall correlation exhibits consistent trends. Both the predicted and experimental values clearly show increased removal efficiencies with an increase in anolyte recirculation and longer HRTs. Several factors, including deviations in reactor design, possible overestimation of the turbulence in certain reactor regions when considering recirculation, the experimental nonuniform biofilm distribution, operational variability, and the complex composition of real wastewater, collectively contribute to deviations between the theoretical predictions and the actual experimental results. These factors represent a limitation of the current modeling approach, highlighting opportunities for refinement in future model developments.

### 3.4 Practical Implications and Future Directions

The findings from this study emphasize the critical role of reactor hydrodynamics in external mass transfer, substrate distribution, and overall MEC efficiency. The identification of flow inefficiencies, such as stagnant regions near reactor walls, corners, and cassette surfaces, highlights the need for reactor designs that promote more uniform flow and substrate delivery to the electroactive biofilms. These dead zones not only reduce substrate removal efficiency but also directly impact bioelectrochemical performance. In these regions, limited substrate transport leads to reduced electron donor availability at the biofilm, which in turn lowers current density and hydrogen production.

To further illustrate the importance of mass transport limitations at pilot scale, the diffusion‐limited current density for acetate was estimated. Considering the inlet substrate concentration (0.4 kg m^−3^), acetate diffusivity (1.26 × 10^−9^ m^2^ s^−1^), and a diffusion boundary layer of 1 mm, the maximum achievable current density is approximately 5 A m^−2^. Thicker boundary layers expected in pilot‐scale systems further reduce current density to 2–3 A m^−2^. These values are significantly lower than typical current densities at lab scale (10–100 A m^−2^), highlighting that external mass transfer, rather than biofilm kinetics, becomes the limiting factor in larger MECs.

Practical modifications such as an increase in recirculation flow, strategic placement of baffles, and optimization of cassette spacing are potential strategies to reduce dead zones and improve mixing. These changes could substantially increase the substrate removal efficiency, as evidenced by the improved performance observed with the recirculation strategy explored here.

Furthermore, increasing the volumetric electrode surface area (m^2^ electrode/m^−3^ reactor) available for biofilm growth and ensuring that this surface is easily accessible to incoming substrates is fundamental. This involves exploring either alternative reactor designs with new stack configurations or 3D anode assemblies. In fact, several studies have suggested the integration of 3D electrodes, such as graphite granules (Li et al. 2013) or granular activated carbon (Liu et al. 2020; Wu et al. 2019), although the costs of these materials are still considered to be high (e.g., granular activated carbon 2000–2500 € ton^−1^) for large‐scale applications. New advancements are being made in the modeling/design (Hernández‐García et al. 2020; Lacroix et al. 2021) and cost‐effective manufacturing of these novel electrodes (Baş and Kaya 2022; Bian et al. 2018; Huggins et al. 2015).

Lastly, the ability to predict and optimize reactor performance using CFD models, as demonstrated in this study, can facilitate this transition by providing insights into large‐scale system behavior without the need for costly experimental trials. Nonetheless, an opportunity for optimization lies in incorporating more complex interactions (e.g., biofilm dynamics and microbial competition), which could offer a more comprehensive understanding of how these systems operate on a large scale. Real municipal wastewater contains a complex mixture of organic compounds, even though acetate was selected as a representative substrate due to its central role as an intermediate in anaerobic degradation pathways and its direct utilization by electroactive microorganisms. This simplification allows the decoupling of hydrodynamic and transport effects from substrate complexity. However, it is acknowledged that this approach may limit the direct quantitative applicability of the results to real wastewater systems, and future models should consider more complex substrate compositions by adding a more diverse microbial composition, including anaerobic fermenters of complex organic substrates to VFAs. The synergy between the different populations and potential biofilm stratification will also be dependent on fluid hydrodynamics.

## 4 Conclusions

This study demonstrates that hydrodynamics and external mass transfer are the dominant factors governing the performance of pilot‐scale cassette‐type MECs. The results show that nonuniform flow distribution leads to dead zones and preferential pathways, limiting substrate availability to electroactive biofilms and reducing reactor efficiency. The implementation of a recirculation flow significantly enhances mass transfer, increasing substrate removal efficiency while maintaining the volumetric treatment capacity. The effectiveness of this strategy depends on optimizing flow rate and its distribution. In contrast, improvements in intrinsic reaction kinetics have a negligible effect under the simulated conditions. The CFD framework developed in this study provides a practical tool for identifying flow limitations and guiding the design of more efficient MEC configurations. Overall, this work contributes to advancing the development of scalable METs for wastewater treatment and resource recovery.

## Author Contributions

**Oscar Guerrero‐Sodric:** conceptualization, formal analysis, investigation, methodology, visualization, writing – original draft, writing – review and editing. **Rholand Jordi Navarro‐Quispe:** data curation, investigation, methodology, software, validation, visualization, writing – review and editing. **Martí Cortada‐García:** conceptualization, formal analysis, methodology, software, supervision. **Juan Antonio Baeza:** conceptualization, formal analysis, investigation, methodology, software, supervision, validation, visualization, writing – review and editing. **Albert Guisasola:** conceptualization, funding acquisition, resources, writing – review and editing, methodology, project administration, supervision, validation.

## Conflicts of Interest

The authors declare that they have no known financial or non‐financial conflicts of interest or personal relationships that could have appeared to influence the work reported in this manuscript.

## Supporting information


**Table S1:** Simulation parameter values.
**Table S2:** Boundary conditions.
**Figure S3:** (A) Shipping container located next to the primary settlers of an urban WWTP (El Prat de Llobregat, Barcelona); (B) Monitoring system and power sources; (C) MEC pilot plant; and (D) Different cassette‐type modules placed inside the reactor.

## Data Availability

The data that support the findings of this study are available from the corresponding author upon reasonable request.
