# Supplementary material for: Computational Fluid Dynamics Reveals Mass Transfer Limitations in a Pilot‐Scale Microbial Electrolysis Cell
Source: Water Environ Res. 2026 Jun 19;98(6):e70452. doi: 10.1002/wer.70452 (PMC13280648; doi:10.1002/wer.70452)
Supplement: Supplementary file 1 — Table S1: Simulation parameter values. Table S2: Boundary conditions. Figure S3: (A) Shipping container located next to the primary settlers of an urban WWTP (El Prat de Llobregat, Barcelona); (B) Monitoring system and power sources; (C) MEC pilot plant; and (D) Different cassette‐type modules placed inside the reactor. [file WER-98-e70452-s001.pdf]

Supplementary information

CFD reveals mass transfer limitations in a pilot-scale microbial electrolysis cell

Oscar Guerrero-Sodric, Rholand Jordi Navarro-Quispe, Martí Cortada-García, Juan Antonio Baeza, Albert Guisasola*

GENOCOV, Department of Chemical, Biological and Environmental Engineering, School of Engineering, Universitat Autònoma de Barcelona, 08193, Bellaterra, Spain

email: Oscar.Guerrero@uab.cat, Albert.Guisasola@uab.cat

** Corresponding author:*
Juan Antonio Baeza
Telephone: 93 581 1587
Email: JuanAntonio.Baeza@uab.cat

S1. Simulation parameters

Table S1: Simulation parameter values.

Model Parameter	Description	Value	References
μ	Water viscosity at 20°C	$1.003 \cdot 10^{-3}$ kg m^{-1} s^{-1}	(Sengers & Watson, 1986)
ρ	Water density at 20°C	998.2 kg m^{-3}	(Sengers & Watson, 1986)
C_μ	Constant for equation 9	0.09	-
C_2	Constant for equation 11	1.9	-
σ_k	Prandtl number for equation 10	1	-
σ_ε	Prandtl number for equation 11	1.2	-
k_r	Kinetic constant for substrate consumption	$4.80 \cdot 10^{-6}$ s^{-1}	-
D_{acet}	Substrate diffusivity	$1.26 \cdot 10^{-9}$ m^2 s^{-1}	(Lide, 2016)
Sc	Turbulent Schmidt number	0.7	-
M_{acet}	Molecular weight of Acetate	59 g mol^{-1}	-
γ	Stoichiometric coefficient	1	-
β	Reaction order	1	-

Table S2: Boundary conditions

Model Parameter	Description	Value
Y_{in}	Inlet acetate fraction	$4 \cdot 10^{-4}$ g_{Acet} g_T^{-1}
u_{in}	Inlet velocity	0.00128 m s^{-1}
Q_{in}	Inlet mass flow rate	0.03322 kg s^{-1}
Q_r	Recirculation mass flow rate	0.01661 - 0.08305 kg s^{-1}
Q_{out}	Outlet mass flow rate	0.03322 kg s^{-1}

S2. MEC pilot plant

The MEC pilot plant (Figure S3) was designed as a modular, cassette-type reactor. The MEC was integrated into an urban wastewater treatment plant (WWTP) and fed with primary effluent. It was inoculated directly with urban wastewater at an applied potential of 1 V. Reactor performance was monitored through regular measurements of COD removal, as well as continuous monitoring of current intensity, hydrogen production, temperature, pH and conductivity. The system consisted of multiple vertically oriented cassette-type cells arranged within a 1 m^3 stainless steel tank, each integrating paired anodic and cathodic chambers separated by anion exchange membranes. The design had an anodic surface-to-volume ratio (~10.9 $m^2\ m^{-3}$), ensuring scalability from the previous prototype while providing a total anodic surface close to 11 m^2. The cathodic compartments were designed to minimize inter-electrode distance and reduce ohmic losses, while optimizing the anodic surface-to-cathodic volume ratio. Hydraulically, the reactor operated under controlled inlet and recirculation flowrates to achieve hydraulic retention times of 1-2 days. The overall configuration aimed to enhance substrate transport, limit solids accumulation through a sloped bottom design, and provide stable operating conditions.

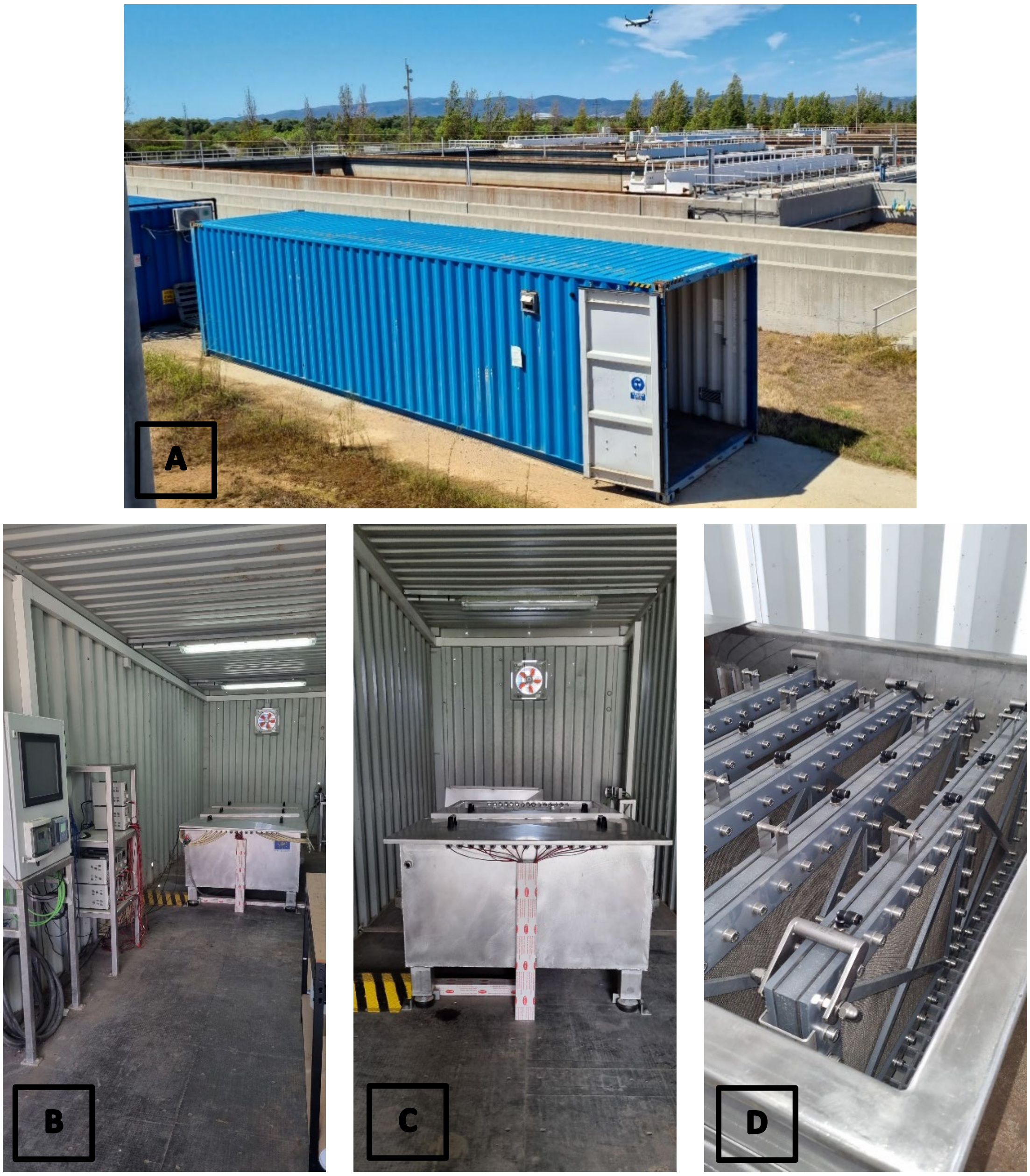

Figure S3 A) Shipping container located next to the primary settlers of an urban WWTP (El Prat de Llobregat, Barcelona); B) Monitoring system and power sources; C) MEC pilot plant; and D) Different cassette-type modules placed inside the reactor.

References

Lide, D. R. (2016). CRC Handbook of Chemistry and Physics, 96th Edition, 2015-2016. *Handbook of Chemistry and Physics*. https://doi.org/10.1136/oem.53.7.504

Sengers, J. V., & Watson, J. T. R. (1986). Improved International Formulations for the Viscosity and Thermal Conductivity of Water Substance. *Journal of Physical and Chemical Reference Data*, *15*(4), 1291–1314. https://doi.org/10.1063/1.555763
